# DNA cytophotometric and histological analysis of *N*-butyl-*N*-(4-hydroxybutyl)nitrosamine-induced precancerous lesions of the bladder urothelium

**DOI:** 10.1007/s00432-016-2153-0

**Published:** 2016-03-31

**Authors:** Hans Helmut Dahm, Claudia von der Haar, Herbert Rübben

**Affiliations:** Institute of Pathology, Hirschlandstraße 97, 73730 Esslingen, Germany; Gynecological Practice, Tiberstraße 7, 48249 Dülmen, Germany; Clinic and Policlinic of Urology, Hufelandstraße 55, 45147 Essen, Germany

**Keywords:** Urothelium, Carcinogenesis, *N*-butyl-*N*-(4-hydroxybutyl)nitrosamine, DNA cytophotometry

## Abstract

**Purpose:**

The morphology of experimentally induced urinary bladder precancerous lesions has been differentially interpreted in the literature. Here, we aimed to describe the development of precancerous lesions of the urothelium histologically and by DNA cytophotometric analysis.

**Methods:**

We induced precancerous lesions of the urothelium in 60 Wistar rats with 0.05 % *N*-butyl-*N*-(4-hydroxybutyl)nitrosamine (BBN) solution as drinking water. After exposure for 2–20 weeks, each animal received tap water for 2 weeks. Subsequently, six animals were killed every 2 weeks, and urothelia of three urinary bladders per time point were examined by DNA cytophotometry of smear preparations. An additional three urinary bladders were processed for histological analysis.

**Results:**

Over 20 weeks, BBN exposure led to a significant difference between the control group and most of the BBN-exposed 2-week groups and to differences between most of these time point groups. After week 4, this difference included a higher proportion of cells with increased nuclear DNA content. At the end of the experiment, DNA cytophotometric values of the urothelium in experimental rats corresponded to those of poorly differentiated urothelial carcinomas.

**Conclusions:**

Biologically significant stages of precancerous lesions were already detectable after 4 weeks of BBN exposure, considerably earlier than previously described in the literature.

## Background

The formal pathogenesis of experimentally induced urinary bladder carcinoma has been differentially described in numerous publications. As in the present work, a 0.05 % *N*-butyl-*N*-(4-hydroxybutyl)nitrosamine solution as drinking water in animal studies was frequently the carcinogen used (Akagi et al. 1973; Arai et al. 1974; Ito et al. 1975; Ito 1976; Kunze et al. 1976; Murphy and Irving 1981; Fukushima et al. 1982; Oliveira et al. 2005, 2007; Palmeira et al. 2009; Vasconcelos-Nobrega et al. 2012). Other researchers have used *N*-(4-(5-nitro-2-furyl)-2-thiazolyl)formamide (Tiltman and Fridell 1971; Cohen et al. 1976), or *N*-methyl-*N*-nitrosourea (Hicks and Chowaniec 1978). Focal or multiple hyperplasias of the urothelium were frequently observed first, while disturbances in stratification and maturation appeared in the hyperplasias after longer exposure periods (Ito 1976; Kunze et al. 1976; Murphy and Irving 1981; Fukushima et al. 1982; Oliveira et al. 2007; Tiltman and Fridell 1971; Hicks and Chowaniec 1978). These changes were designated as mild, moderate, or severe hyperplasia (Cohen et al. 1976), dysplasia, or carcinoma in situ (Oliveira et al. 2005, 2007). The spectrum of observed changes seemed to be identical with those of the human urinary bladder (Oliveira et al. 2007; Palmeira et al. 2009; Tiltman and Fridell 1971). Thus, we aimed to characterize the time-dependent morphological changes that occur during urothelium precancerous lesion development in rats using both histological and DNA cytophotometric analysis.

## Materials and methods

Precancerous lesions were induced in urinary bladder urothelium of 60 female Wistar rats (Landeszuchtanstalt Hannover, Germany) by adding 0.05 ml BBN (Professor Dr. R. Preussmann, German Cancer Research Center, Heidelberg, Germany) to 100 ml of their drinking water. Three animals were held in each Makrolon cage (Ehret, Emmendingen, Germany). At the beginning of the experiment, all rats weighed 180–200 g. Standard feed (Eggersmann, Rinteln, Germany) and BBN solution were provided ad libitum. Room temperature was 22 °C, relative humidity was 55 %, and the light–dark cycle was 12 h. Seven control animals received tap water. Experimental rats were treated with BBN for 2–20 weeks, followed by 2 weeks of tap water provision. Every 2 weeks, six animals were killed under ether anesthesia with a Nembutal overdose applied intraperitoneally (Abbott, Ludwigshafen, Germany). Urothelial smears of three urinary bladders at each time point were prepared and fixed in a methanol–formalin–acetic acid solution (Böhm 1968). The optimal hydrolysis time of Feulgen staining (Pearse 1968) was 45 min at 28 °C in 4 N HCl (Böhm 1968).

After staining with Schiff’s reagent (Graumann 1953), we covered the slides with coverslips using oil as intermedium (Cargille Laboratories, Cedar Grove, USA) and obtained measurements at 40-fold magnification on an M85a scanning microdensitometer (Vickers Instruments, York, UK) interfaced with an HP 9825A computer (Hewlett-Packard, Böblingen, Germany). We used the recommended wavelength of 570 nm for all measurements (Fossa 1975). For the smears of each urinary bladder, we measured 100 cell nuclei by random placement (series A and B) or focused on the 50 largest nuclei of one selected area (series C). Lymphocytes from the same smear served as a reference for diploid DNA values. Measured values were divided into 24 classes with a breadth of 0.5c (0.25c to >11.75c) and analyzed using the Chi-square test.

For comparative histological examination, three animals per time point were perfusion-fixed with buffered formalin–glutaraldehyde mixture (McDowell and Trump 1976). Semi-thin sections (Autocut 1140, Reichert-Jung, Wetzlar, Germany) of urinary bladder cross sections embedded in Technovit 7200 VLC (Haereus Kulzer, Wehrheim, Germany) were stained with hematoxylin–eosin (HE).

## Results

### Histology

**Fig. 1:**
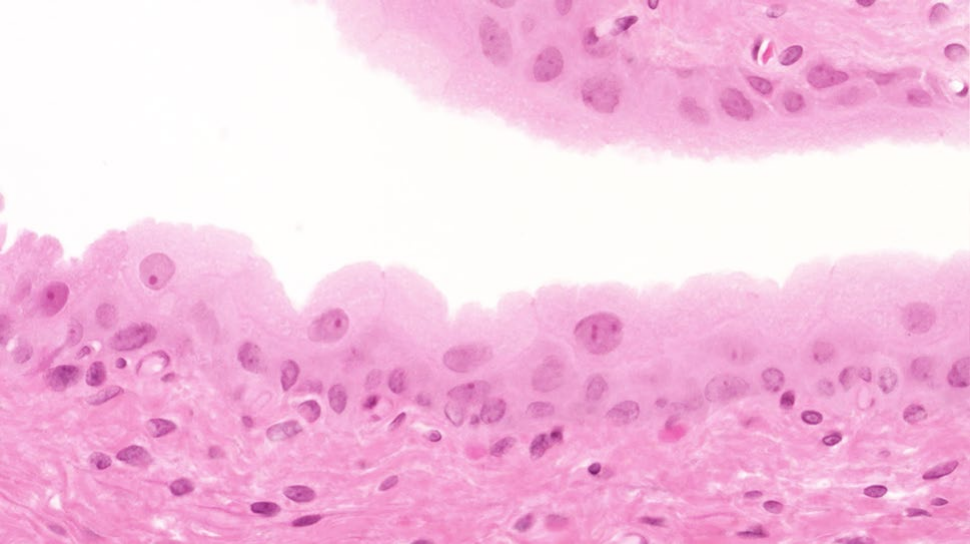
Normal three-layer urothelium (HE, ×400 magnification)

**Fig. 2:**
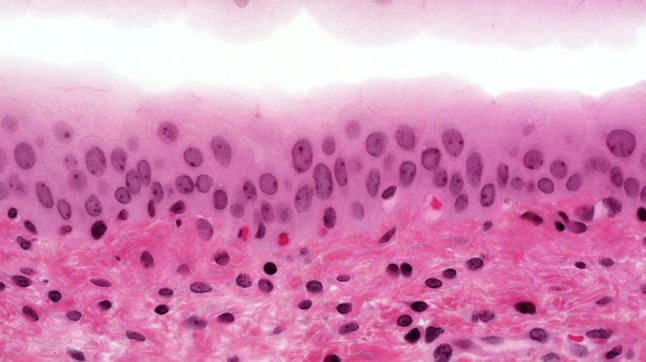
Week 2: hyperplastic urothelium (HE, ×400 magnification)

**Fig. 3:**
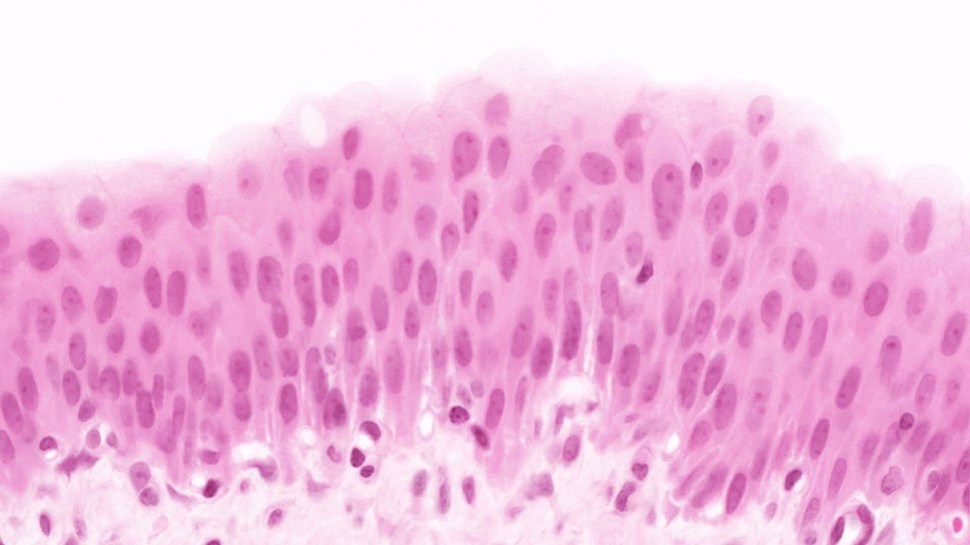
Week 4: increased numbers and nuclear enlargement of intermediate cells (HE, ×400 magnification)

The urothelium of control animals was composed of one layer each of superficial, intermediate, and basal cells (Fig. 1). After 2 weeks of BBN treatment, the urothelium of experimental animals was slightly expanded and displayed increased numbers of basal and intermediate cells (Fig. 2). By week 4 of exposure, the intermediate cells had increased to five layers, their cylindrical nuclei oriented perpendicular to the basement membrane. Enlargement of individual nuclei aroused suspicion of a mild dysplasia (Fig. 3). At week 6, maturation of superficial cells was almost entirely absent. The urothelium was primarily composed of cell layers of varying thickness and appeared dysplastic as most nuclei were mildly to moderately enlarged. We noted mild-to-moderate dysplasia in papillary urothelial proliferations and cornified squamous metaplasia at week 8. The dysplasia foci had more strongly expanded by week 10.

**Fig. 4:**
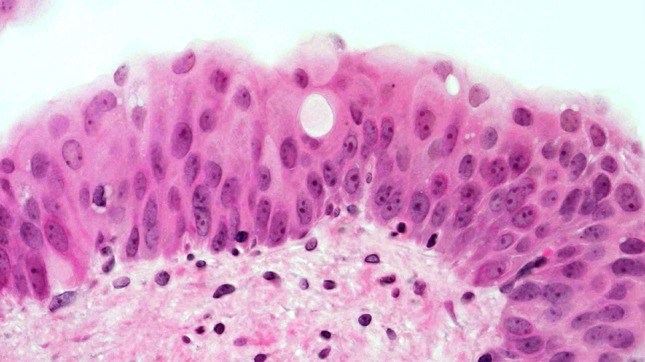
Week 16: moderate-to-severe dysplasia of the urothelium (HE, ×400 magnification)

**Fig. 5:**
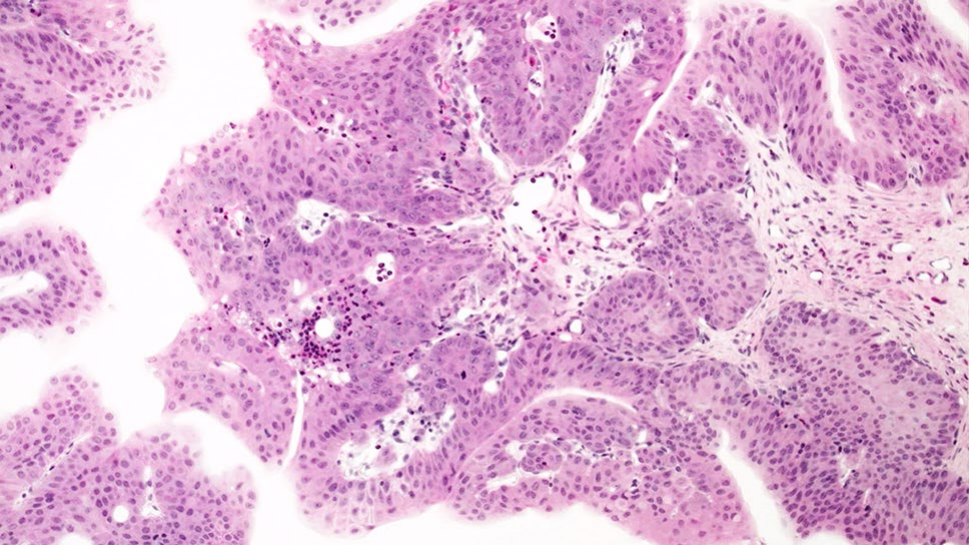
Week 18: papillary tumor with atypical urothelium (HE, ×40 magnification)

At week 12, mild-to-moderate dysplasia predominated the urinary bladders, followed by the appearance of small-focus moderate atypias in urothelium, squamous metaplasia, and papillomatous tumors at week 14. At week 16, a moderate diffuse dysplasia of the urothelium and papillary tumors prevailed (Fig. 4). We observed severe nuclear anaplasia from weeks 18 to 20 (Fig. 5) and could not distinguish the complex proliferations of epithelia and stroma in the inverted papillary tumors from an invasive carcinoma by the conclusion of the experiment.

### DNA cytophotometry

Throughout the 20-week trial, DNA cytophotometric measurements of cells from most of the experimental rats were significantly different from those in the control group. In addition, measurements of most of the 2-week experimental group significantly differed from each other (*p* < 0.05).

**Fig. 6:**
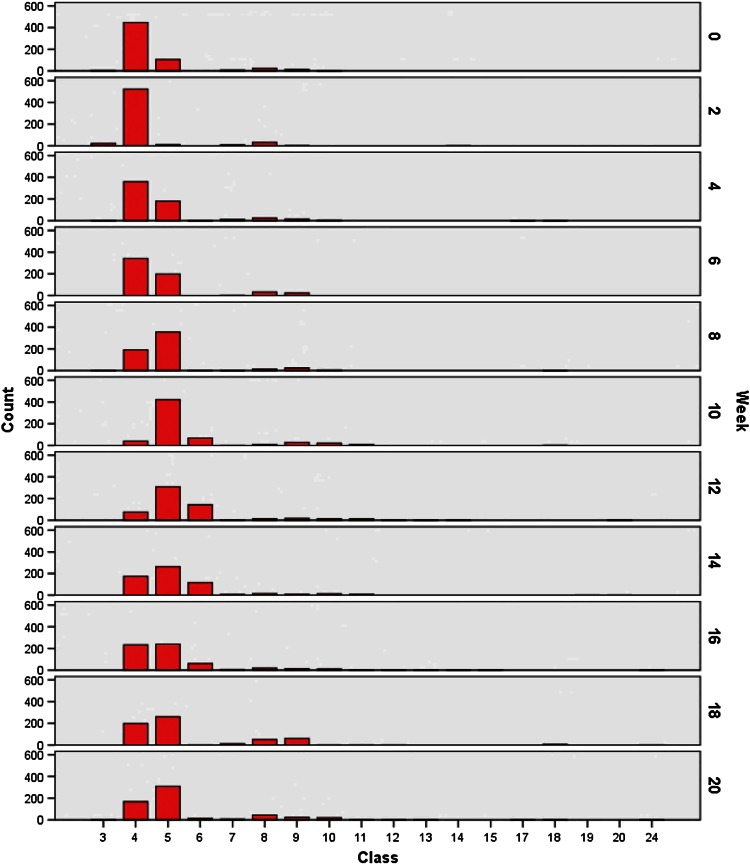
Histogram, series A and B

**Fig. 7:**
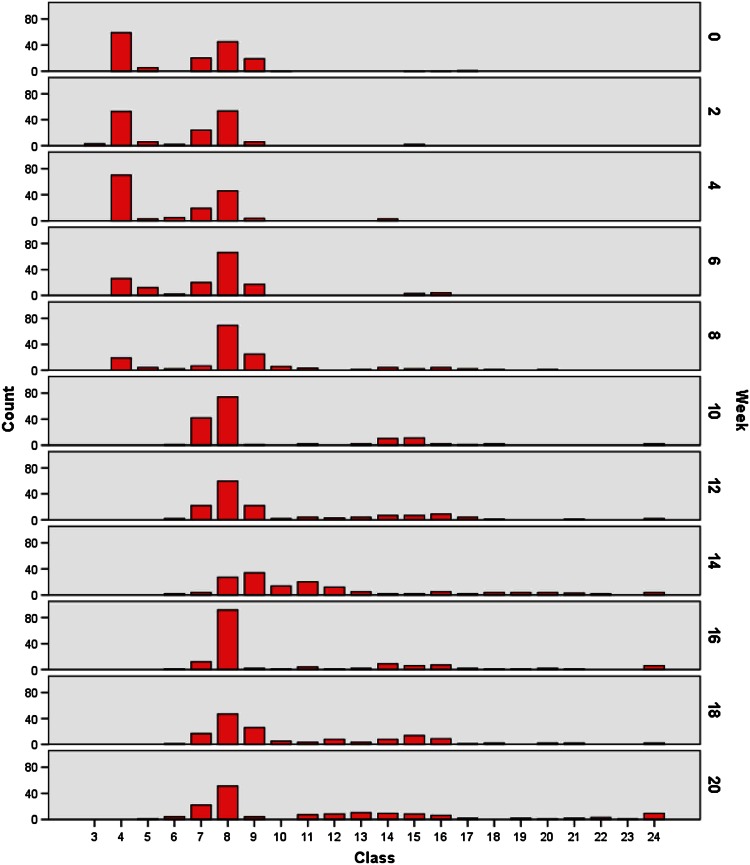
Histogram, series C

Resulting measurements and adapted comparative values of the random samples (A and B) are summarized in Table 1 and Fig. 6, while those of the selective sample (*C*) are summarized in Table 2 and Fig. 7. At week 2, we observed decreased values at 2.5c and 4.5c in favor of values at 1.5c and 2c and a peak at 3c. At week 4, values at 2.5c increased again, and atypical values appeared at 7c and 9c. Tetraploid values had also increased and were broadly dispersed by week 6, as well as the appearance of DNA values in the octoploid region with higher frequency. Week 8 yielded atypical values at 7c and 10c and peaks at 3c and 5c. Values at 5c and 7c had increased at week 10. From weeks 10 to 20, numerous additional values appeared at 3c, 6c, and 8c, and by week 20, 9 of 150 nuclei exhibited values in the >11.75c class.

**Table 1:** DNA values, series A and B

Class	*C*	*W*0	*W*2	W4	*W*6	*W*8	*W*10	*W*12	*W*14	*W*16	*W*18	*W*20
1	0.25–0.75											
2	0.75–1.25											
3	1.25–1.75	13	22	2		3						1
4	1.75–2.25	1036	522	359	344	191	40	76	175	234	199	168
5	2.25–2.75	247	11	179	198	354	422	309	262	240	261	310
6	2.75–3.25			1		5	68	143	116	60	2	14
7	3.25–3.75	19	10	12	2	2	1	5	6	6	12	8
8	3.75–4.25	51	32	25	32	14	8	14	13	20	52	44
9	4.25–4.75	31	2	15	24	24	28	18	7	13	62	24
10	4.75–5.25	3		5		6	22	15	12	11	2	20
11	5.25–5.75						9	13	7	4	1	4
12	5.75–6.25							2		3	1	1
13	6.25–6.75							1		2		1
14	6.75–7.25		1					2		3		
15	7.25–7.75									2		
16	7.75–8.25											
17	8.25–8.75			1								1
18	8.75–9.25			1		1	2				6	2
19	9.25–9.75								1			
20	9.75–10.25							2	1			
21	10.25–10.75											
22	10.75–11.25											
23	11.25–11.75											
24	>11.75									2	2	2

**Table 2:** DNA values, series C

Class	*C*	*W*0	*W*2	*W*4	*W*6	*W*8	*W*10	*W*12	*W*14	*W*16	*W*18	*W*20
1	0.25–0.75											
2	0.75–1.25											
3	1.25–1.75		3									
4	1.75–2.25	137	53	70	26	19						
5	2.25–2.75	12	6	3	12	4						1
6	2.75–3.25	0	2	5	2	2	1	2	2	1	1	4
7	3.25–3.75	47	24	19	20	7	42	22	4	12	17	22
8	3.75–4.25	105	54	46	66	69	74	60	27	92	47	51
9	4.25–4.75	44	6	4	17	25	1	22	34	2	26	4
10	4.75–5.25	1	0	0	0	6	0	2	14	1	5	
11	5.25–5.75					3	2	4	20	4	3	7
12	5.75–6.25							3	12	1	8	8
13	6.25–6.75					1	2	4	5	2	3	10
14	6.75–7.25			3		4	10	7	2	9	8	9
15	7.25–7.75	1	2		3	2	11	7	2	6	14	8
16	7.75–8.25	1			4	4	2	9	5	7	9	6
17	8.25–8.75	2				2	1	4	2	2	1	2
18	8.75–9.25					1	2	1	4	1	2	0
19	9.25–9.75								4	1		2
20	9.75–10.25					1			4	2	2	1
21	10.25–10.75							1	3	1	2	2
22	10.75–11.25								2			3
23	11.25–11.75											1
24	>11.75						2	2	4	6	2	9

## Discussion

Exposure to BBN induces selective tumor formation in urinary bladder urothelia in numerous species (Druckrey et al. 1964), including our chosen test model, which has been comprehensively reviewed previously (Vasconcelos-Nobrega et al. 2012). To better characterize these BBN-induced precancerous changes, we analyzed DNA values of the epithelial cell nuclei cytophotometrically in parallel with histological observations and noted many significant time-dependent developmental features.

Normal urothelium was comprised of three cell layers with primarily diploid DNA values (2c). After 2 weeks of BBN exposure, we observed mild urothelial hyperplasia but without significant changes of the DNA values. At week 4, nuclei of intermediate cells became enlarged, and hyperdiploid DNA values increased. At week 6, histological changes consistent with dysplasia were accompanied by increased tetraploid and octoploid DNA values. Foci with a slight to moderate and, finally, great nuclear enlargement were found during the following weeks, with increasing numbers and to an increasing degree. We noted varying amounts of atypical tri-, hexa-, and octoploid DNA values between weeks 10 and 20, and by week 20, papillary tumors and severe nuclear anaplasia of the urothelium were present, together with numerous nuclear DNA values in the >11.75c class.

The morphology of experimentally induced precancerous lesions of the urinary bladder has been interpreted differently throughout the literature due to a lack of a consistent classification system. In addition, detailed cytological analyses are limited (Murphy and Irving 1981). Common histological changes observed after BBN exposure include polyploid, papillary, or solid focal hyperplasias after 8 weeks (Ito 1976); hyperplasias, papillomas, and carcinoma appearance after 12 weeks BBN followed by 28 weeks tap water (Ito et al. 1975) and 20 weeks BBN (Akagi et al. 1973); and focal hyperplasias with few atypias that were interpreted as precancerous lesions as well as formation of papillary transitional cell carcinomas after 20 weeks of BBN exposure (Arai et al. 1974). Large numbers of papillary hyperplasias and papillomas with few atypias or in situ carcinomas, and individual invasive tumors have all been observed after 12 weeks BBN followed by 21 weeks tap water (Kunze et al. 1976). Many hyperplasias demonstrated considerable nuclear polymorphism (Hicks and Chowaniec 1978).

Only recently DNA cytophotometric examinations of experimentally induced urinary bladder tumors have been described (Oliveira et al. 2005, 2007; Palmeira et al. 2009). For example, after 4 weeks of BBN exposure, hyperplasias with 22.2 % aneuploid nuclei and dysplasias with 55.5 % aneuploid nuclei were found in paraffin sections from mice (Oliveira et al. 2005).

The general level of aneuploidy can, like the degree of proliferation, be determined by flow cytometry at relatively low cost (Gustafson and Tribukait 1985) but with a minimum of 10,000 cells per probe (Tribukait et al. 1979). One limitation of this approach is that it only separates low-grade from high-grade bladder tumors that are designated as diploid or aneuploid (Tribukait et al. 1982).

DNA image cytometry has the advantage of a higher sensitivity over flow cytometry on cell populations with small aneuploid peaks (Palmeira et al. 2008).

This high resolution could be maintained even with grade 1 urothelial carcinomas by improving the precision of measurements and the introduction of sensitive statistical tests to identify DNA-aneuploid stemlines. This DNA-aneuploid stemline interpretation system yielded a sensitivity of 76.3 % and a specificity of 100 % when applied to biopsy specimens of 60 grade 1 urothelial carcinomas and 50 biopsy specimens of normal bladder mucosa (Planz et al. 1996).

Like the interpretation of histological findings, that of nuclear DNA values of experimental bladder tumors is also not standardized. The normal urinary bladder epithelium of the rat is polyploid and comprised of diploid cells (2n) with 42 chromosomes (Oliveira et al. 2007), tetraploid (4n) cells, and a few octoploid (8n) cells (Cooper et al. 1969). The nuclear DNA content during cell cycle phase G1 is given a euploid or diploid DNA value (2c). Throughout S phase, the diploid DNA content doubles and may range from 2c to 4c (Kiefer and Sandritter 1976). In the normal urothelium, 1.7 % of cells are in *S* phase, while nearly 20 % of cells of poorly differentiated carcinomas are in *S* phase (Farsund et al. 1984). In laboratory animals, tetraploid cells can likewise divide after appropriate cellular damage (Cooper et al. 1969). Although the DNA content of individual cells in normal tissue may deviate from average values, it is typically stable. Dispersed or elevated DNA values indicate regeneration or a malignant process (Leuchtenberger et al. 1954), so DNA cytophotometric analysis cannot consistently distinguish between neoplasia and cellular regeneration (Murphy et al. 1986). The DNA content of well-differentiated urothelial tumors is similar to that of normal diploid cells (Fossa 1975), while cells of moderately and poorly differentiated bladder carcinomas are usually aneuploid (Gustafson and Tribukait 1985). Our unpublished measurements yielded average values of 2.4c for G1 transitional cell carcinomas (*n* = 34), 3.9c for G2 tumors (*n* = 34), and 6.6c for G3 tumors (*n* = 22).

Representative (Table 1; Fig. 6) and selective (Table 2; Fig. 7) surveys of random samples in our current study revealed remarkably different findings. For example, only few pathological DNA values >6c (class 12) of series C were represented in series A and B.

Increased DNA values between 2c (class 4) and 4c (class 8) at week 4 could be attributed to an elevated number of S-phase cells. However, at this time point increase in values at 4.5c to 5c and the occurrence of values at 7c and 9c were noteworthy. In addition, from week 4 on a relatively high peak at 2.5c had developed and remained till week 20, possibly representing a near-diploid cell line of low-grade dysplasia. The relative increase in values in the tetraploid range (4c, class 8) was striking at week 6, as these DNA values corresponded to those of a G2 carcinoma or moderate dysplasia. Values between 4c and 8c at week 8, together with values beyond 11.75c, and peaks at 3c (class 6) at week 10 also suggested a neoplastic process. From week 10 on, DNA values in peaks or a broad unimodal distribution corresponded to those of G3 carcinomas.

Histologically, we observed epithelial hyperplasia with slightly increased numbers of basal and intermediate cells at week 2. By week 4, however, the uniformity of cells and isolated atypical nuclei already indicated an early intraepithelial neoplasia or mild dysplasia. The increased near-diploid and tetraploid DNA values at week 4 provided no clarification (Murphy et al. 1986), particularly as we could not directly correlate DNA content with cellular morphology. However, our determination of mild dysplasia was supported by the occurrence of atypical values at 7c and 9c as well as by the results of Oliveira et al. (2005). At week 6 and afterwards, morphological characteristics and DNA values were evidence to suggest the development of intraepithelial neoplasias that temporally increased in severity. Our findings indicated that, in an increasing number of cells, specific changes in nuclear DNA content occurred at random after appropriate exposure to BBN.

The test model presented here has been used recently in genetic and molecular pathological studies, leading to the characterization of *p53* and *H*-*Ras* mutations, elevated values of epidermal growth factor receptor, loss of alleles, and chromosomal alterations during early, non-invasive stages of precancerous lesion development (Vasconcelos-Nobrega et al. 2012). Thus, future analyses of multiple genetic alterations of the bladder urothelium now may be performed on a valid morphological basis.
